# Expected costs and benefits of genetic counselling and germline genetic testing in metastatic prostate cancer

**DOI:** 10.1007/s00439-026-02854-1

**Published:** 2026-08-29

**Authors:** Michiel Vlaming, Lambertus A. L. M. Kiemeney, Wouter Koole, Inge M. van Oort, Eveline M. A. Bleiker, Margreet G. E. M. Ausems, Geert W. J. Frederix

**Affiliations:** 1 Department of Genetics, Division Laboratories, Pharmacy and Biomedical Genetics, University Medical Center Utrecht, Utrecht, The Netherlands; 2 Department of Urology, Radboud university medical center, Nijmegen, The Netherlands; 3 Science Department IQ Health, Radboud university medical center, Nijmegen, The Netherlands; 4 Department of Urology, University of Groningen, University Medical Center Groningen, Groningen, The Netherlands; 5 Division of Psychosocial Research and Epidemiology, The Netherlands Cancer Institute, Amsterdam, The Netherlands; 6 Department of Clinical Genetics, Leiden University Medical Center, Leiden, The Netherlands; 7 Department of Clinical Genetics, The Netherlands Cancer Institute, Amsterdam, The Netherlands; 8 Department of Epidemiology and Health Economics, Julius Center for Health Sciences and Primary Care, University Medical Center Utrecht, Heidelberglaan 100, Utrecht, 3584 CX The Netherlands

## Abstract

Identifying pathogenic germline variants in men with metastatic prostate cancer is important for therapeutic options and for identifying relatives who may have a high cancer risk. To keep genetic testing costs manageable, it is important to identify which patients should be selected for testing. In this study, we compared expected costs and number of identified pathogenic variants in two scenarios: offering genetic testing to all men with metastatic prostate cancer versus testing only those with additional risk factors linked to a higher likelihood of carrying a pathogenic variant. Costs were evaluated with a mainstream genetic testing pathway, where testing is discussed by a non-genetic healthcare professional. Using Dutch national incidence data, predefined healthcare cost frameworks, and a hypothetical assumption for the acceptance rate of patients, we modelled total costs for the Netherlands and number of identified pathogenic variants for each approach. The costs of genetic testing and post-test counselling decreased threefold when applying selection criteria, compared to the scenario where all metastatic prostate cancer patients are eligible. However, applying selection criteria would result in missing 41% clinically relevant pathogenic germline variants that would otherwise be identified. This implies that, at national level, a discussion should be started regarding willingness to pay for identifying pathogenic germline variants.

## Introduction

Germline genetic testing is recommended in several international guidelines for all patients with metastatic prostate cancer (mPCa) regardless of additional selection criteria to guide additional therapeutic options (Lowrance et al. 2023; National Comprehensive Cancer Network 2025). Studies reported between 3 and 19% of these patients to carry a pathogenic germline variant in a cancer predisposition gene (Abdi et al. 2022; Abida et al. 2017; Antonarakis et al. 2018; Cheng et al. 2023; Greenberg et al. 2021; Hart et al. 2016; Isaacsson Velho et al. 2018; Ledet et al. 2021; Mandelker et al. 2017; Mijuskovic et al. 2018; Petrovics et al. 2019; Priestley et al. 2019; Pritchard et al. 2016; Robinson et al. 2015). Relatives may also carry the same pathogenic variant, and be at increased risk of developing cancer (Antoniou et al. 2003; Breast Cancer Association Consortium 2021; Kuchenbaecker et al. 2017; Li et al. 2022; Page et al. 2019; Tai et al. 2007). Pathogenic germline variants, identified with germline genetic testing, in mPCa are primarily found in the genes *BRCA2*, *ATM* and *CHEK2* (Abdi et al. 2022; Abida et al. 2017; Antonarakis et al. 2018; Cheng et al. 2023; Greenberg et al. 2021; Hart et al. 2016; Isaacsson Velho et al. 2018; Ledet et al. 2021; Mandelker et al. 2017; Mijuskovic et al. 2018; Petrovics et al. 2019; Priestley et al. 2019; Pritchard et al. 2016; Robinson et al. 2015). Such variants are significantly associated with an elevated risk of breast, ovarian, and pancreatic cancer in female relatives and, in case of *BRCA2*, with both breast and prostate cancer in male relatives (Antoniou et al. 2003; Breast Cancer Association Consortium 2021; Kuchenbaecker et al. 2017; Li et al. 2022; Page et al. 2019; Tai et al. 2007). Female relatives carrying a pathogenic variant in one of these genes can opt for breast surveillance. Relatives with a pathogenic *BRCA1* or *BRCA2* variant can opt for a risk-reducing mastectomy (Carbine et al. 2018; Federatie Medisch Specialisten/Richtlijnendatabase 2025; Ludwig et al. 2016; National Comprehensive Cancer Network 2026; Wood et al. 2020). For women with those variants, risk-reducing salpingo-oophorectomy is recommended (Federatie Medisch Specialisten/Richtlijnendatabase 2025; Ludwig et al. 2016). PSA screening is recommended for men with a pathogenic germline variant in *BRCA2*, and may be considered in men with pathogenic variants in other cancer predisposition genes (National Comprehensive Cancer Network 2026; Page et al. 2019).

We previously performed a cohort study in the Netherlands, in which 779 patients received germline genetic testing through a mainstreaming pathway. In such a pathway, genetic testing is discussed and requested by the treating physician (urologist, oncologist) or nurse (specialist nurse, nurse practitioner), i.e., a non-genetic healthcare professional (Vlaming et al. 2022). In this study, we found that 5.9% of unselected mPCa patients carried a pathogenic germline variant in a high-risk cancer gene *BRCA1*, *BRCA2* and *PALB2* or moderate-risk cancer gene *ATM* and *CHEK2* (Vlaming et al. 2025). Other genes potentially associated with prostate cancer, such as the MMR genes and *HOXB13*, were not routinely included in the gene panel test. Several risk factors were associated with the presence of a pathogenic germline variant. These risk factors were “being diagnosed with another primary cancer”, “Jewish ancestry”, “first- or second-degree relative with breast cancer < 50 years” and “first- or second-degree relative with pancreatic cancer”. The predicted probability of a pathogenic variant in patients with one of these risk factors was 7.9% – 18.6%. Over the years, germline panels have expanded, genetic testing costs have decreased significantly and guidelines have started using broader selection criteria (Lowrance et al. 2023; National Comprehensive Cancer Network 2025). Currently, there is no threshold of identified pathogenic variants for adopting a germline genetic test, but the diagnostic yield should be proportionate to the associated costs.

Despite the benefits of germline genetic testing, the growing adoption of these tests leads to substantial increases in genetic counselling and testing costs, resulting in questions about affordability and value for money. Debate is currently ongoing about determining the most effective way of selecting prostate cancer patients for germline genetic testing (Loeb et al. 2025). Although no formal threshold is used for the percentage of pathogenic variants required to justify genetic testing, it is highly questionable whether a 5.9% likelihood of identifying such pathogenic variants is sufficient to recommend genetic testing for all mPCa patients. It is particularly debatable, given that the majority of identified pathogenic variants (68%) were found in moderate-risk cancer genes, which typically have a lower medical impact on relatives compared to high-risk cancer genes (Wood et al. 2020), and are therefore clinically less relevant.

Narrowing indications for germline genetic testing could result in cost savings, but would likely also result in a decrease of the total number of identified pathogenic variants. Early economic evaluations could give insight into the likely impact of stricter indications on both identified pathogenic variants and costs. These insights are based on various assumptions and can further inform decision-making early on consequences of implementing the testing strategy of choice.

In the current study, we aimed to compare the efficacy of two scenarios of eligibility criteria for germline genetic testing in a mainstream genetic testing pathway: scenario 1, in which all patients with mPCa are eligible for genetic testing, and scenario 2, in which only patients with mPCa at increased risk of carrying a pathogenic germline variant due to one or more established risk factors are eligible.

## Methods

### Study procedure

Data in this study were collected from a multicentre (*n* = 15) cohort study conducted in the Netherlands (Vlaming et al. 2022). Patients in this study were included through a mainstream genetic testing pathway. The non-genetic healthcare professional gathered key information about the patient’s personal cancer history and family cancer history using a checklist on paper or integrated into the electronic medical record (Vlaming et al. 2025). All patients with mPCa (TxNxM1) were eligible for germline genetic testing, regardless of their medical history or family background.

The genetic test result was communicated by letter to the patient. For patients with a pathogenic germline variant in *BRCA2*, *BRCA1* or *PALB2*, cascade screening is offered for all first-degree relatives of a carrier with a pathogenic germline variant, without any additional requirements (Grutters and Christiaans 2024). When finding a variant in *CHEK2* however, predictive genetic testing of relatives is only offered in the Netherlands if it is clinically relevant, meaning there is a family history of breast cancer (in a first- or second-degree relative). For *ATM*, predictive testing is only offered when a first- or second-degree relative has a history of breast or pancreatic cancer.

Patients without a pathogenic germline variant, but with a relevant family history of ovarian cancer or multiple pancreatic cancers could also be referred to the genetics department (also referred to as “complex genetic counselling”). This pathway was used in three centres (*n* = 246 patients, 32%); in the other twelve centres additional referrals were not registered. The rationale for the additional referrals, particularly in these family histories, was to assess within this post-test counselling session whether affected relatives were eligible for germline genetic testing of relevant high-risk genes. We calculated the percentage of patients who were referred to the genetics department (*n* = 10 out of 246) in those three centres and extrapolated this percentage to the entire cohort.

### Patient population

The annual number of synchronous mPCa patients (i.e., no previous localized prostate cancer) in the Netherlands was obtained from the Netherlands Cancer Registry (NCR), maintained by the (Netherlands Comprehensive Cancer Organisation (IKNL), 2026). It was based on the 5-year average from 2018 to 2022 (*n* = 3,623 per year). The extra annual number of patients with metachronous mPCa (first diagnosis of localized prostate cancer and metastases during follow-up) in the Netherlands is not yet clear and therefore not publicly available. Based on personal communication with the principal investigator of the Dutch prostate cancer registry “CAPRI”, unpublished data showed that approximately 29.5% of mPCa cases in the Netherlands were metachronous, and 70.5% were synchronous. Given 3,623 new synchronous mPCa patients per year, this corresponded to an estimated 1,516 metachronous mPCa patients annually, resulting in a total of approximately 5,139 new mPCa cases annually. In total, 20.4% of the total number of patients with mPCa had an additional risk factor (recalculated from previous data) (Vlaming et al. 2025), which summed up to an average of 739 patients with synchronous mPCa per year and 309 with metachronous mPCa – in total, 1,048 patients annually.

### Healthcare costs related to genetic counselling and testing

The healthcare costs of genetic counselling and testing in the Netherlands are structured within a predefined framework known as Diagnosis Treatment Combinations (DBCs) (Kroneman et al. 2016). A DBC includes the average total costs of all steps within a healthcare pathway, so there is no billing for individual components of the pathway. Healthcare insurers reimburse hospitals for the cost of a DBC. When a patient was a carrier of a pathogenic germline variant, he received post-test genetic counselling at the genetics department, also referred to as “complex genetic counselling”. In 2025, the fixed price for the DBC related to complex genetic counselling is €1,578 (Dutch Healthcare Authority, NZa 2025). The price for germline genetic panel testing is set at €1,621 in 2025.

### Costs per pathogenic germline variant

**Fig. 1:**
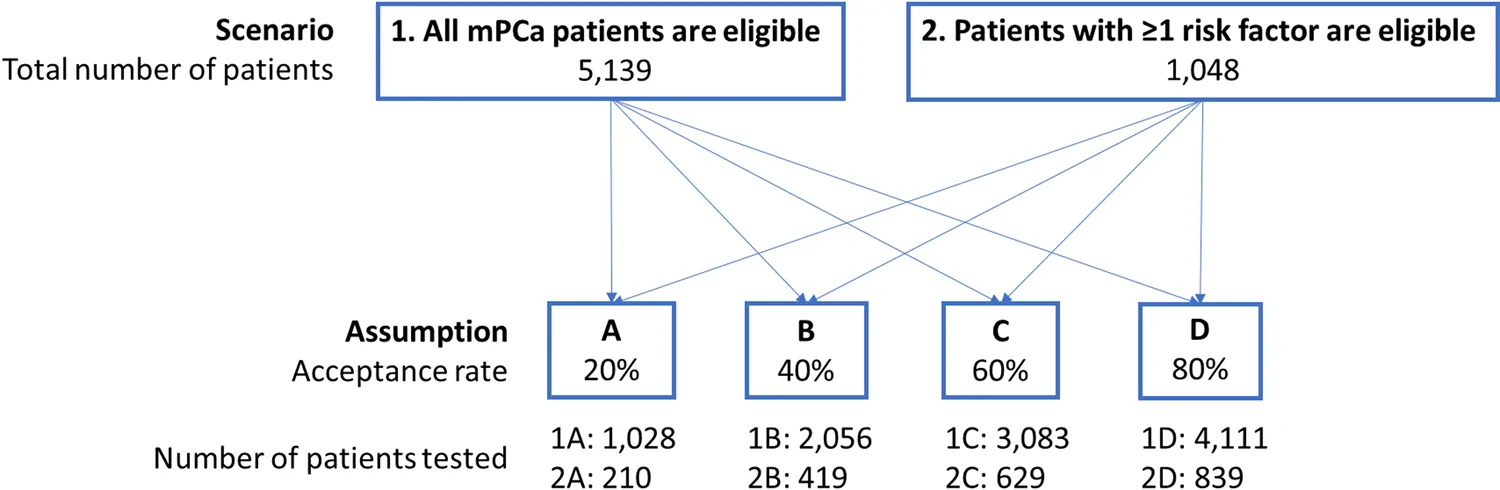
Overview of used scenarios and assumptions

**Fig. 2:**
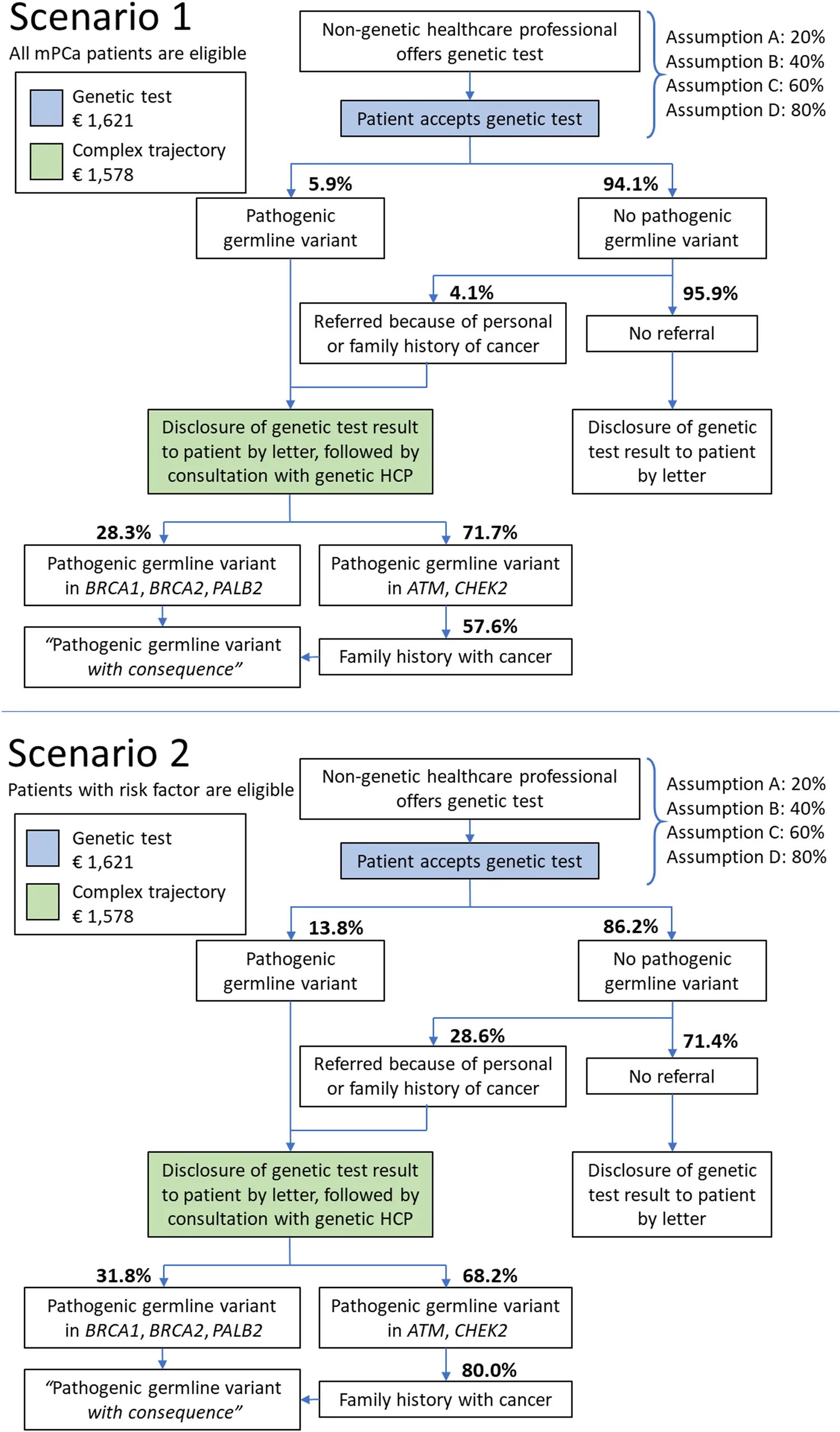
Decision tree with transition probabilities based on data by Vlaming M. et al. (Vlaming et al. 2025) when **A** all metastatic prostate cancer patients are eligible (100%) or **B** only metastatic prostate cancer patients with risk factor are eligible (20.4%)

To estimate the costs of genetic testing per identified pathogenic germline variant, we compared two scenarios. In scenario 1, all patients with mPCa were eligible for genetic testing and in scenario 2, there was a preselection of patients with one or more previously established risk factors of carrying a pathogenic germline variant (Vlaming et al. 2025). These risk factors were: “being diagnosed with another primary cancer diagnosis”, “Jewish ancestry”, “first- or second-degree relative with breast cancer < 50 years” and “first- or second-degree relative with pancreatic cancer”. We extrapolated data on the percentage of carriers from the Dutch cohort study to the entire Dutch patient population with mPCa (Vlaming et al. 2022). Family history of prostate cancer or ovarian cancer was not a significant predictor of carrying a pathogenic germline variant in our previous study (Vlaming et al. 2025) and was therefore not included in the current analyses. Considering that not all patients may opt for, or be offered genetic testing, we used several assumptions based on different acceptance rates (20%, 40%, 60% and 80%) to gain insight into the potential effect (see Fig. 1). Using a simplified decision tree we calculated both outcome (number of identified pathogenic variants) and total costs for both scenarios with all acceptance rates (Fig. 2).

### Identified variants in pathogenic germline variants

By completing the decision tree in Fig. 2, we calculated the number of pathogenic germline variants in high-risk and moderate-risk cancer genes identified for each acceptance rate in both scenarios. By subtracting the number of identified variants in scenario 2 from the total identified variants in scenario 1, we determined how many variants were missed due to the selection criteria. Although those numbers differed across the acceptance rates, the relative proportions remained constant, allowing us to calculate the percentage of missed variants.

## Results

We calculated the total costs of genetic testing and post-test genetic counselling for all acceptance rates across scenario 1 and 2. Within scenario 1, where all patients with mPCa were eligible for genetic testing, the annual costs of genetic testing ranged between €1,597,766 and €6,391,066, depending on the acceptance rate that was used, shown in Table 1. Of the patients undergoing genetic testing, 61 to 243 would be diagnosed with a pathogenic germline variant in *BRCA1*, *BRCA2*, *CHEK2*, *ATM*, or *PALB2*, based on the prevalence reported in the previous Dutch study (Vlaming et al. 2025). Additionally, 39–157 patients would be referred to the Genetics department due to a relevant family history, despite not having a pathogenic germline variant. Combining these patients with the ones with a pathogenic variant would result in an additional cost of €151,342 to €605,366 for post-test genetic counselling. The combined total healthcare costs would therefore range between €1,749,108 and €6,996,432 for this cohort of patients. In all assumptions for scenario 1 the total healthcare costs per identified pathogenic variant would be €28,820.

**Table 1:** Costs and patient numbers associated with most and least conservative assumption across different scenarios

	1. All patients with mPCa eligible for germline genetic testing		2. Patients with mPCa and ≥ 1 risk factor eligible for germline genetic testing	
	Assumption 1 A	Assumption 1D	Assumption 2 A	Assumption 2D
Acceptance rate	20%	80%	20%	80%
Patients undergoing genetic testing (n)	1,028	4,111	210	839
Costs of genetic testing	€1,597,766	€6,391,066	€339,968	€1,359,874
Identified gPVs (n)	61	243	29	116
Identified gPVs in high-risk cancer genes (n)	17	69	9	37
Identified gPVs in moderate-risk cancer genes (n)	44	174	20	79
Referred patients without gPV (n)	39	157	52	206
Costs of genetic counselling	€151,342	€605,366	€127,268	€509,071
Total costs	€1,749,108	€6,996,432	€467,236	€1,868,945

*gPV* germline pathogenic variant, *mPCa* metastatic prostate cancer

When only patients with a predefined risk factor were eligible for genetic testing, the number of eligible patients significantly decreased. Costs and diagnostic yield could be recalculated accordingly. The total costs of genetic testing and counselling for scenario 2 varied between €467,236 and €1,868,945, depending on the acceptance rate used (Table 1). Using these predefined risk factors as selection criteria would decrease the total costs by €1,281,872 to €5,127,487, both being a decrease of 73% compared to scenario 1. The costs per identified pathogenic germline variant would total up to €16,103.

**Fig. 3:**
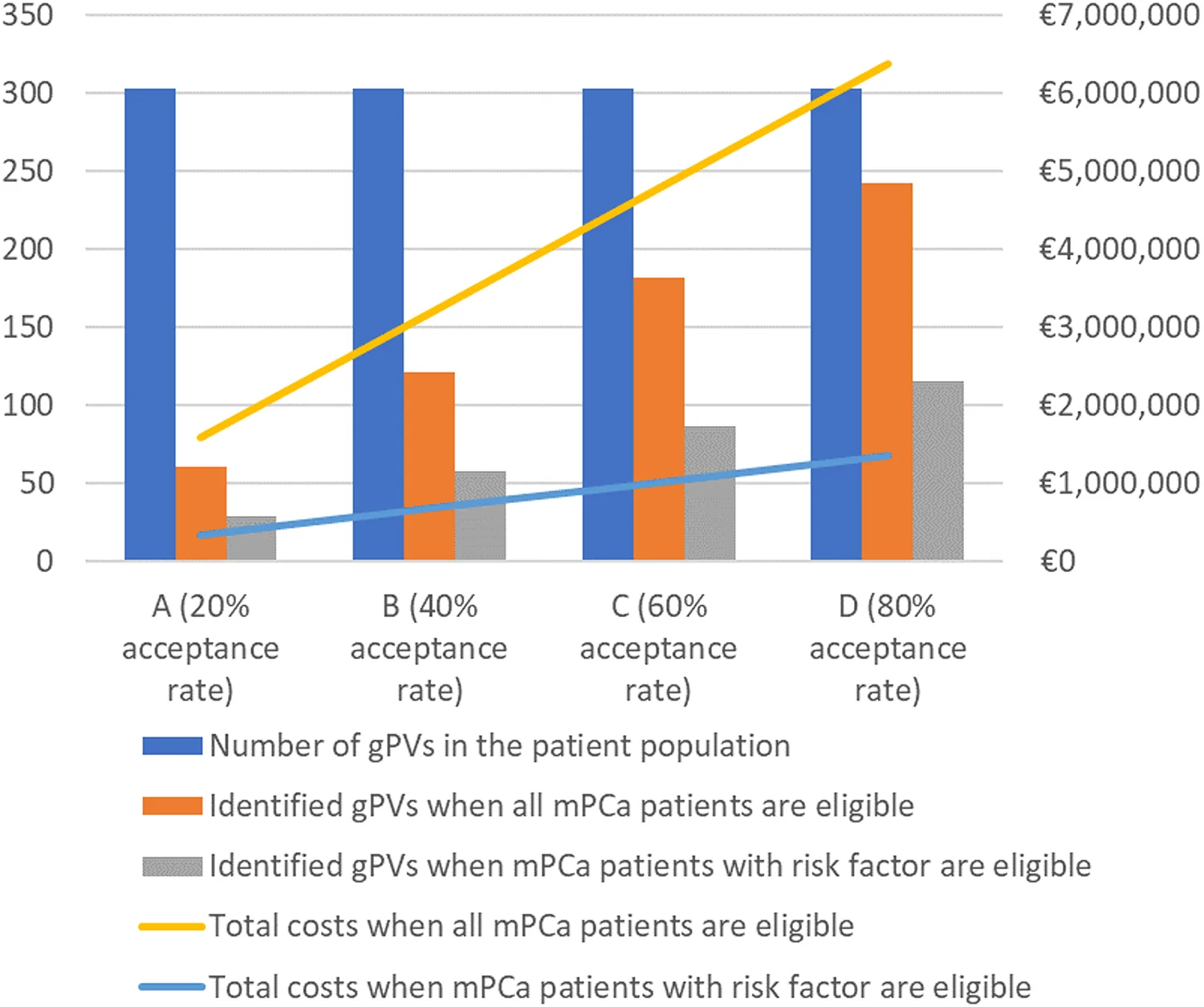
Difference between expected germline pathogenic variants in patient population, identified germline pathogenic variants and associated costs. gPV: germline pathogenic variant, mPCa: metastatic prostate cancer

In Fig. 3, the relationship between the number of gPVs in the patient population, the identified gPVs and total costs across the different scenarios and acceptance rates is shown. Across each acceptance rate, using the predefined selection criteria, would lead to 52% of patients with a pathogenic variant not being identified since they did not meet the selection criteria. Using the selection criteria, 46% of gPVs in high-risk genes would be missed and 55% of all gPVs in moderate-risk genes. Of all patients with a pathogenic germline variant, 69% of patients had a pathogenic variant that could have consequences within the family (i.e., pathogenic germline variant in (1) *BRCA2*, *BRCA1*, *PALB2* or (2) *CHEK2*, *ATM* with relevant family history). When only patients with a predefined risk factor were assessed, this percentage increased to 86%. However, when using the risk factors as selection criteria, 41% of pathogenic variants that might have consequences in the family would be missed compared to the scenario with no selection criteria.

## Discussion

To our knowledge, this is the first study that examined the relationship between selection criteria for germline genetic testing, expected genetics-related healthcare costs and prevalence of identified pathogenic variants in patients with mPCa. We showed that, within our study population, narrowing the selection criteria to four established risk factors (“being diagnosed with another primary cancer”, “Jewish ancestry”, “a first- or second-degree relative with breast cancer < 50 years” and “a first- or second-degree relative with pancreatic cancer”) reduces costs by 73%. However, this approach would result in missing 52% of pathogenic germline variants that would otherwise be identified. This implies that a balanced decision should be made weighing both costs and outcomes, informed by this analysis.

The largest cost difference came from genetic testing itself, which could be reduced by 79% by applying the additional selection criteria. Savings from post-test genetic counselling would amount to 16% across all acceptance rates. Given that genetic testing is by far the largest cost item, optimizing selection criteria is crucial to balancing cost reduction with identified pathogenic variants. By optimizing selection criteria, we could reduce the number of tests conducted, and lower costs while still identifying as many pathogenic germline variants as possible.

A decrease of 52% of identified pathogenic variants suggested that the current selection criteria were far from optimal, i.e., that patients with pathogenic variants in mPCa often lacked additional risk factors. When considering only pathogenic variants that might have consequences within a family, the percentage of missed variants dropped to 41%, though this still represents a significant loss. The potential harm of missing variants in moderate-risk cancer genes without a family history of breast or pancreatic cancer is debatable, as carriers in such families in the Netherlands currently do not face a significant change in cancer prevention strategies compared to the general population (Federatie Medisch Specialisten/Richtlijnendatabase 2025). However, minimizing missed pathogenic variants in high-risk genes, 46% of which were missed with selection criteria, or moderate-risk genes with a relevant family history is crucial.

According to the NCCN and AUA guidelines, broad selection criteria are used for germline genetic testing, since all patients with mPCa, and even high-risk localized prostate cancer are eligible for testing (Lowrance et al. 2023; National Comprehensive Cancer Network 2025). The latest European EAU guideline recommends germline genetic testing in mPCa patients only if they are candidates for targeted treatment (Cornford et al. 2024). It is known that guideline-based testing in prostate cancer, based on the 2019 NCCN criteria, misses up to 42% of pathogenic variants (Shore et al. 2023). Therefore, currently there are initiatives that advocate germline genetic testing in all patients with prostate cancer irrespective of stage (Tuffaha et al. 2023). Both our study and the previously mentioned articles contribute to evaluating the trade-off, at country level, between maximizing the identification of clinically relevant pathogenic germline variants and the associated willingness to pay for identifying such variants.

As an alternative to germline genetic testing, patients with mPCa may undergo tumour genetic testing. If a pathogenic variant is detected through tumour genetic testing, germline genetic testing is necessary to determine whether the variant is of germline or somatic (non-hereditary) origin (Li et al. 2020). A key advantage of tumour genetic testing is its ability to identify more variants, some of which may be essential for determining additional treatment options (Longoria et al. 2024). For instance, in case of pathogenic variants in the DNA damage repair pathway, up to half of the variants detected in tumour genetic testing are of somatic origin (Lang et al. 2019) and are therefore not identified through germline genetic testing. Since more pathogenic variants are identified in the same number of patients, tumour genetic testing might be more cost-effective, although exact data on this subject is missing. The high number of identified pathogenic variants underlies the recommendation for tumour genetic testing in all major guidelines in metastatic prostate cancer (Tuffaha et al. 2023). However, there is a trade-off: patients with a pathogenic variant in the tumour will often require germline testing as well, to determine the consequences for relatives. This significantly increases costs, and for variants in certain genes (e.g., *ATM* and *BRCA2*), the majority of patients with an abnormal tumour genetic test will have a normal germline test (Li et al. 2020; Truong et al. 2023). The costs of tumour genetic testing vary widely; however, one study estimated a price of €1,538 per Next Generation Sequencing test, which is somewhat comparable to the Dutch cost of a germline test (€1,621) (Grossman et al. 2026). When both somatic and germline genetic testing are required for patients with a detected somatic variant, overall costs will increase substantially. In addition, tumour genetic testing misses around 7% of pathogenic germline variants (Terraf et al. 2022). Additional prospective research is needed to evaluate the real costs and diagnostic yield of tumour genetic testing and the associated germline analyses, as well as to determine the most effective approach for integrating tumour and germline genetic testing, since this has not yet been investigated.

### Strengths and limitations

This study provides novel insights into the costs of genetic testing in mPCa and the consequences of narrowing inclusion criteria, within a mainstream genetic testing pathway. We combined both study data and real world data, and used various plausible acceptance rates to account for uncertainties. While the healthcare system in the Netherlands may differ from those in other countries, the findings are still relevant and helpful for countries facing similar challenges, particularly those with limited capacity of genetic healthcare professionals. Moreover, we aimed to ensure, by transparently providing insights into all input parameters, that our study can easily be replicated with other input parameters by choice.

A limitation was that we had to rely on assumptions for the percentage of patients who were offered and accepted genetic testing, since this data was not available for the Netherlands. There is a substantial variation between the most and least conservative estimates, which leads to a significant difference in costs for genetic testing as well. Another uncertainty is the proportion of patients referred to post-test counselling who actually attend. However, no reliable data are available to determine this number. Given the already considerable variability in the assumptions regarding patients who are offered and accept genetic testing, it is not feasible to introduce a separate set of assumptions for this parameter.

Another limitation was that we only included the genetics related healthcare costs (e.g. genetic testing and complex genetic counselling). The costs of genetic testing and complex genetic counselling were the only costs that were clearly defined, but other potential costs remained undefined. For example, there are undefined costs associated with pre-test genetic counselling by non-genetic healthcare professionals. When a pathogenic variant is identified, genetic testing can be performed in relatives and they can undergo preventive measures or intensified screening to prevent cancer or detect cancer at an early stage (Carbine et al. 2018; Ludwig et al. 2016; Page et al. 2019; Wood et al. 2020). This could lead to downstream cost savings in the future and, consequently, increase “willingness to pay” of healthcare payers. For female carriers of a pathogenic *BRCA1/2* variant, preventive measures such as risk-reducing surgeries are cost-effective in terms of costs per quality-adjusted-life-year (QALY) (Petelin et al. 2020; Schrauder et al. 2019). Additionally, cost-effectiveness has been demonstrated for germline *BRCA* testing of men with mPCa when cascade testing of male and female first-degree relatives of *BRCA*-positive patients are taken into account (Teppala et al. 2024).

The data used in this analysis comes from a single-cohort study, which means that generalisability might be limited. In addition, not all variables that we used as risk factors for pathogenic germline variants have been confirmed as such in mPCa by other research groups. Therefore, further validation of these risk factors, that were previously mentioned, is necessary in diverse populations of patients with mPCa.

In this study, we focused on pathogenic germline variants in *BRCA1*, *BRCA2*, *ATM*, *CHEK2* and *PALB2*. We acknowledge that including other genes in the gene panel could potentially lead to different results between the two scenarios and the associated costs.

Lastly, the assessment of the percentage of referrals for patients without a pathogenic variant but with a relevant family history was based on data from only three out of fifteen hospitals, covering 246 study participants (32%). No data on this topic could feasibly be retrieved from other hospitals, or no such pathway existed in those hospitals.

## Conclusion

Narrowing selection criteria for germline genetic testing from all patients with metastatic prostate cancer to only patients with metastatic prostate cancer and an additional risk factor decreases the combined costs of genetic testing and genetic counselling considerably. However, within our study population this strategy leads to missing 41% of clinically relevant pathogenic variants in *BRCA1*, *BRCA2*, *PALB2*, *CHEK2* or *ATM*, which could have consequences within a family. Based on this, decision-makers must balance the benefits of identifying pathogenic germline variants against the associated willingness to pay for identifying those variants.

## Data Availability

The data supporting the findings of this study are available upon reasonable request.
